# Synthesis Route, Microstructural Evolution, and Mechanical Property Relationship of High-Entropy Alloys (HEAs): A Review

**DOI:** 10.3390/ma14113065

**Published:** 2021-06-04

**Authors:** Omoyemi Temitope Onawale, Prince Valentine Cobbinah, Rivel Armil Nzeukou, Wallace Rwisayi Matizamhuka

**Affiliations:** Department of Metallurgical Engineering, Vaal University of Technology, Andries Potgieter Boulevard, Vanderbijlpark 1911, South Africa; prinzcobbs@gmail.com (P.V.C.); rivelnzeukou@yahoo.fr (R.A.N.); wallace@vut.ac.za (W.R.M.)

**Keywords:** high-entropy alloy, powder metallurgy, melting and casting, microstructural evolution, additive manufacturing, solid solution strengthening

## Abstract

Microstructural phase evolution during melting and casting depends on the rate of cooling, the collective mobility of constituent elements, and binary constituent pairs. Parameters used in mechanical alloying and spark plasma sintering, the initial structure of binary alloy pairs, are some of the factors that influence phase evolution in powder-metallurgy-produced HEAs. Factors such as powder flowability, laser power, powder thickness and shape, scan spacing, and volumetric energy density (VED) all play important roles in determining the resulting microstructure in additive manufacturing technology. Large lattice distortion could hinder dislocation motion in HEAs, and this could influence the microstructure, especially at high temperatures, leading to improved mechanical properties in some HEAs. Mechanical properties of some HEAs can be influenced through solid solution hardening, precipitation hardening, grain boundary strengthening, and dislocation hardening. Despite the HEA system showing reliable potential engineering properties if commercialized, there is a need to examine the effects that processing routes have on the microstructure in relation to mechanical properties. This review discusses these effects as well as other factors involved.

## 1. Introduction

The discovery and application of alloying and composite technology have made possible the achievement of various categories of materials that exhibit a wide range of properties. An example is a novel alloy system known as high-entropy alloys (HEAs). Yeh, et al. [1] defined HEAs, by composition, as alloys having at least five principal elements, wherein each has a concentration between 5 and 35 at.%. In line with Yeh et al.’s research and definition, Miracle et al. [2] also categorized HEAs based on elemental composition and configurational entropy.

Some categories of the HEAs studied are lanthanide HEAs [3,4], refractory HEAs (RHEAs) [5], platinum group metal HEAs (PGM-HEAs) [6], and lightweight HEAs (LWHEAs) [7]. Lanthanide HEAs consist of lanthanide (4f) elements, such as Gd, Lu, Tb, Dy, and Tm [4]. Nb, Ta, W, Mo, V, and Hf usually make up RHEAs [8]. RHEAs are primarily developed for exceptionally high-temperature applications (up to 1400 °C), but with a disadvantage of high density. PGM-HEAs consist of precious elements (Au, Ag, Pt, Ir, Os, and Re), while LWHEAs are composed of low-density elements such as Li, Mg, Be, and Al.

Over the past decade, material scientists have used several techniques in synthesizing HEAs, such as the melting and casting route, the powder metallurgy (PM) route, and additive manufacturing (AM) processing techniques. The melting and casting route is the most common and relatively cheaper fabrication route. However, the high tendency of a heterogeneous structure with elemental segregation and defects accompanies the melting and casting route [9]. The PM process involving mechanical alloying (MA) and consolidation by spark plasma sintering (SPS) is usually used in attempts of achieving homogeneous microstructures in HEAs. The MA and SPS processes are quick, material efficient, and energy efficient. Nonetheless, contamination from the grinding media poses a challenge for the PM synthesis of HEAs [10]. In contrast, the AM fabrication route in recent years has received more attention in circumventing the flaws of other synthesis processes. The AM process is a flexible manufacturing technique with the capability of producing parts with complex geometries, finer microstructures, mass customization, and efficient material usage [11].

Four core effects influence the microstructural evolution of HEA systems. They are the high-entropy effect, sluggish diffusion effect, lattice distortion effect, and cocktail effect. The high-entropy effect indicates that both configurational entropy and enthalpy play significant roles in the solid solution formation of HEAs. Furthermore, the formation of an intermetallic and solid solution chiefly depends on the entropy of mixing (ΔS_mix_), the enthalpy of mixing (ΔH_mix_), and the atomic size difference (δ) [12,13]. Sluggish diffusion explains the strengthening attribute of HEAs [14]. Moreover, a fine precipitate and a controlled grain structure are usually formed as a result of the sluggish diffusion effect. In contrast, the severe lattice distortion effect in HEAs results from the random arrangement of different sizes of atoms (making up the alloy) distributed in a crystal lattice [15,16]. The effect suggests that the pair distribution function directly relates to the distribution of the interatomic spacing on a local atomic level [15]. The “cocktail” effect indicates the possibility of achieving unexpected properties in an HEA system from mixing different elements in a chosen proportion [17]. Hence, resulting properties from this HEA are usually expected to surpass individual elemental properties that make up the system [18,19]. The properties of HEAs are known to be a result of the overall contributions of the constituent phases influenced by phase shape, phase distribution, and boundaries, as well as the properties of each phase [20]. The cocktail effect ranges from the atomic-scale, multi-element composite effect to the microscale and multiphase composite effect [20].

There is no doubt that the basis of HEA design revolves around these so-called core effects. Hence, most HEAs studied have been derived from these basic principles [21,22,23]. Nevertheless, the validity of these core effects has been doubted by some researchers recently.

To date, the microstructural evolution of HEAs is still not well understood. This makes the prediction of processing–structure relationships quite a challenge. The design approach adopted by most researchers does not follow a specific logic; rather, a number of these alloys are a result of a trial-and-error approach. Although attempts have been made to categorize these alloys according to the intended application, there still exists a multitude of alloys exhibiting a wide range of properties. It is against this background that this review attempts to unravel some processing techniques used so far in synthesizing HEAs. This paper will also try to establish a structure–property relationship and link it to the processing route used.

## 2. Microstructural Evolution of HEAs Synthesized through the Melting and Casting Route

**Figure 1:**
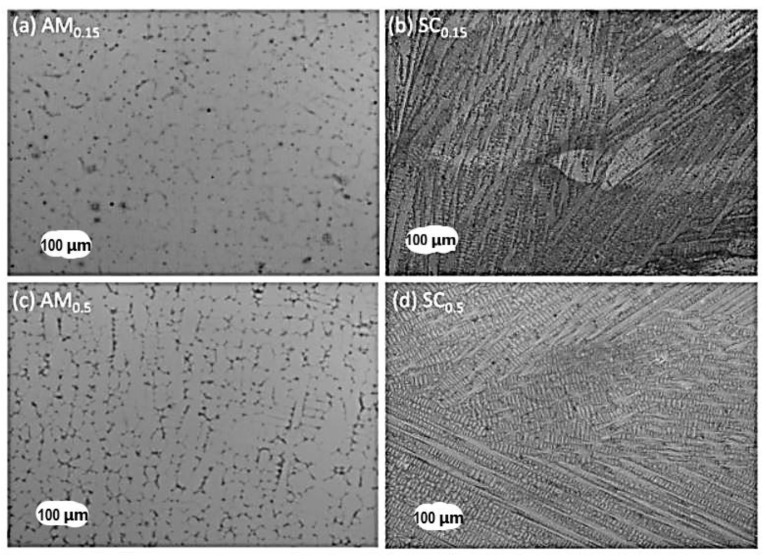
OM micrographs of arc-melting (AM*_x_*) and suction-casting (SC*_x_*) alloys (x = 0.15 and 0.5). (**a**) Columnar cellular structure and (**c**) non-equiaxed columnar dendrite by arc-melting; (**b**) and (**d**) columnar dendrite grains by suction casting [29].

HEAs have been fabricated using the melting and casting route. Table 1 is a compilation of some HEAs fabricated using the melting and casting route. In general, the melting and casting route is a liquid-state processing route with equilibrium or non-equilibrium cooling rates. An advantage of processing HEAs using the melting and casting route is the high temperatures that can be realized or needed to melt some elements that make up the HEA alloy [9]. Melting and casting can be achieved by a tilt casting furnace or suction casting. During the melting and casting process, the phase transformation of HEAs occurs during solidification (cooling). During solidification, phase evolution depends on the collective mobility or distribution of constituent elements making up the alloy [9]. However, the rate of cooling, differences in the local atomic arrangement, and the varying elemental diffusivity can influence the solid phase that is first to form and the microstructure of the alloys [24,25]. HEAs fabricated using the melting and casting route usually show dendritic microstructures with interdendritic segregations. For instance, AlCoCrFeNi HEAs fabricated using the melting and casting route have been shown to exhibit BCC + B2 phases with dendritic microstructures [26,27]. Tian et al. [28] studied the effect of different cooling rates using arc-melting processing routes in the fabrication of AlCoCrFeNi HEAs. Both studies observed nanoparticles of the B2 phase within the grains of the single-phase BCC structure. Lv et al. [29] compared the effect of cooling rates on the microstructure of Al_x_CoCrFeNi HEAs using arc-melting and suction casting. The higher cooling rate of the suction casting resulted in refined columnar dendrite grains, while the arc-melting process led to a columnar cellular structure (see Figure 1). However, both processes led to the formation of BCC and FCC phases, with the inclusion of a B2 phase for arc-melting and Laves phases for suction casting. Thus, the melting and casting techniques with faster cooling rates favor the formation of a more dominant single phase and limit the precipitation of secondary phases [30]. Several studies have reported the cooling rate effects on HEAs fabricated using melting and casting [25,31,32].

From another perspective, HEA phase formation during fabrication via the melting and casting route is suggested to hinge on binary constituent pairs rather than individual constituent elements [75,76]. An HEA system such as the AlCoCrFeNi alloy forms a BCC structure after processing; although among the constituent elements, only Cr and Fe have BCC crystal structures. The AlNi pair, from the possible binaries in the AlCoCrFeNi system, serves as the primary crystal structure in the AlCoCrFeNi HEA. This is due to the similar lattice parameter between AlNi (0.28810 nm) and AlCoCrFeNi (0.289675 nm) [77,78]. In addition, AlNi has the largest negative enthalpy of formation among all the binary pairs in AlCoCrFeNi [79,80,81]. The AlNi binary pair stabilizes over a wide composition field from 1638 °C down to room temperature and can dissolve other constituent elements [82,83]. The other elements, therefore, dissolve into the primary lattice due to their chemical compatibility and mixing entropy effect [84]. During solidification, Cr having the highest melting point is the first element to solidify. Cr remains segregated from the liquid mixture up to 1350 °C at the equiatomic composition [85,86]. In contrast, Al has the lowest melting temperature and thus has the highest diffusivity during solidification. The effect of Al addition on 3d transition metal-based HEAs such as AlCoCrFeNi been studied [87,88,89]. The increasing quantity of Al promotes the formation of the BCC phase [89,90,91]. Moreover, Wang et al. [92] and Rogström et al. [93] observed that the AlCoCrFeNi HEA exhibits a spinodal microstructure of an A2 ((Cr, Fe)-rich) disordered solid solution and a modulated B2 ((Al, Ni)-rich) ordered solid solution. The A2 phase forms at temperatures below 600 °C, while the B2 phase forms at higher temperatures [92].

Some examples of HEAs that exhibit a single-phase FCC structure after melting and casting are the CoCrFeMnNi HEA structure [14,94], the Al_x_CoCrCuFeNi alloy system [95,96], the CoCrCuFeNi HEA [97,98], the FeCoNiCrCuO_0.5_Al_x_ HEA [49], and the Al_x_CoCrFeNiTi_y_ HEA [51,99]. The binary constituents in these HEAs encourage the formation of the FCC phase. In addition, the addition of elements such as Cu and Ti stabilizes the FCC phase [100,101]. In the Al_x_CoCrFeNi alloy system, the addition of Ti promotes phase evolution from the BCC to an FCC phase [81]. Furthermore, when Al in AlCoCrFeNi is replaced with Cu to form the CoCrCuFeNi alloy, the FCC phase forms instead of an A2 + B2 structure associated with AlCoCrFeNi. CuCo, CuNi, CoNi, FeNi, and CoFe, which make up the binary constituents in the CoCrCuFeNi alloy, all have an FCC structure and promote the FCC phase. In addition, the use of Mn to form CoCrFeMnNi also leads to a single-phase FCC structure [102].

## 3. Powder Metallurgy

### 3.1. Mechanical Alloying (MA)

MA is a solid-state process that allows the dispersion of insoluble phases and the addition of reactive alloying elements to produce composite metal powders with controlled microstructures. In this process, a high-energy stirred ball mill or shaker mill is used to subject blended powders of known particle size to a compressive force to agglomerate the powders. The mechanical alloying process can be grouped into five different stages: the initial stage, a period of welding, an equiaxed particle formation period, the start of random welding orientation, and steady-state processing [103]. Hence, these periods can be explained in terms of [103] (a) the distribution of the powder and shape, (b) how hard the material is on the ball surface, (c) the microstructure of the powder and material on the ball surface, and (d) the material division between ball surfaces and free powders. The formation of composite particles and the refinement of structure occur over time as a result of repeated welding and fracturing of free powder particles.

MA has been given more preference in the literature recently compared to conventional methods such as melting and casting. It does aid the homogeneous distribution of particle size, consolidation, and a reduction in grain size and helps in proper densification of elemental powders [104]. The mechanical energy input as well as the rate of work hardening among the material influences the rate of structural refinement in HEAs. These thus have a positive effect on the resulting mechanical properties of HEAs. Hence, the MA technique is considered a more convenient and cost-effective way of synthesizing nano-crystalline materials with a uniform microstructure [104].

### 3.2. Spark Plasma Sintering (SPS)

SPS uses a direct current (DC) pulse voltage and current during compaction of as-milled alloy powders to produce a bulk alloy in the solid state [105]. Spark plasma and spark impact pressure are used to generate high temperatures between the particles, causing melting of the surface of the particles during the sintering process [106]. Chakraborty et al. [107] classified the mechanism of sintering of a bulk alloy using SPS into five stages: generation of plasma, heating, melting, sputtering of the molten particles, and neck growth. The as-milled powder is directly charged into a graphite die through which current and uniaxial force or pressure are applied simultaneously, resulting in a fully dense material with outstanding mechanical properties [108]. Densification is achieved for both conductive and nonconductive powders in a short time due to the fast heating rate [106]. Application of high heating and cooling rates in SPS enhances densification and promotes the diffusion mechanism, which helps to maintain the powders’ intrinsic properties in a fully dense state [108]. A higher cooling rate reduces the level of micro-segregation in the alloy and leads to refinement of grain size, which is achievable in SPS [109]. Hence, the need for heat-treating SPS-fabricated HEAs might be unnecessary. This is in contrast with the as-cast microstructure having the possibility of generating a dendritic microstructure during the solidification process [8]. Therefore, SPS present tremendous potential and can sinter a fully dense and nearly single-phase alloy with a refined microstructure from elemental powder. The microstructure and phases in the final sintered products are significantly influenced by the processing parameters.

**Figure 2:**
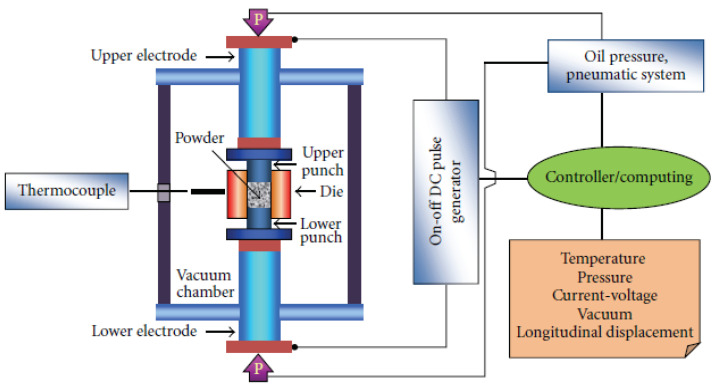
Schematic diagram of the SPS process [108].

The SPS method is widely used, especially to synthesize nano-crystalline microstructures, advanced ceramics, and composite materials, owing to its advantages over conventional sintering techniques [110]. The sintering system not only offers ease of operation but also results in less grain growth in the microstructure. Hence, this leads to an improved microstructure, further leading to better resulting mechanical properties when compared to the melting and casting route. Solid solution strengthening is achieved through this fabrication technique, which also has a positive impact on the mechanical properties of HEAs. These are some of the reasons many researchers lately prefer SPS as a mode of compacting powders, in addition to its significant advantages such as shorter processing time [110], flexible sintering temperature, avoidance of porosity, easy control of sintering parameters, and energy-efficient processing. Figure 2 shows a schematic diagram of the SPS process.

### 3.3. Microstructural Evolution of HEAs Synthesized Using Powder Metallurgy

The PM synthesis route through MA and consolidation by SPS has been used in the fabrication of HEAs [10,111,112]. The MA and SPS techniques are solid-state processing routes mostly used to achieve nanocrystalline HEAs [113]. The MA process is a non-equilibrium process and thus leads to the formation of metastable phases. Table 2 shows a summary of evolved phases of some HEAs fabricated through MA and SPS.

**Figure 3:**
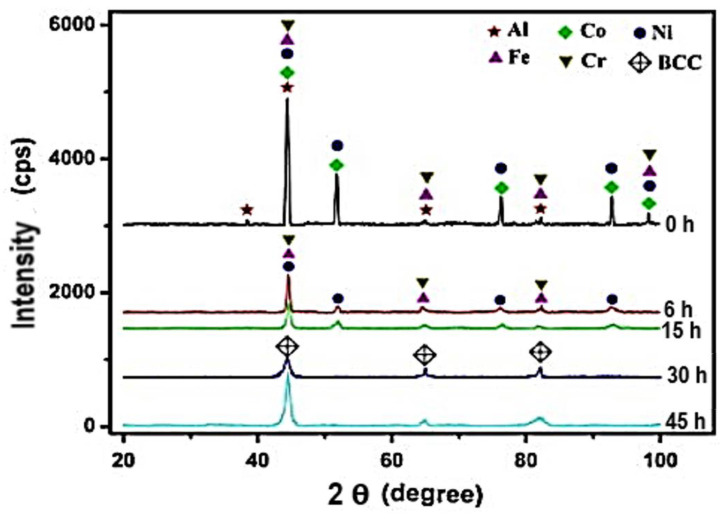
XRD pattern of FeNiCrCo_0.3_Al_0.7_ with different milling times [114].

The binary-phase diagrams of constituent elements and the thermodynamic concept of mixing enthalpies in an HEA system help understand or predict possible phases during MA [125]. The energy involved during the MA process generates heat and influences the phases that form. Typically, an alloy system would tend toward phases for which free energy is the lowest. So, for the desired phase(s), it is imperative to achieve the right balance of parameters used in the MA process, such as milling duration, ball-to-powder ratio (BPR), and the grinding media. Chen et al. [114] studied the alloying behavior, microstructure, and mechanical properties of FeNiCrCo_0.3_Al_0.7_ HEA fabricated using the MA and SPS route. The FeNiCrCo_0.3_Al_0.7_ HEA formed a refined and homogeneous supersaturated BCC solid solution after 45 h of milling. After the first 6 h of milling, Al and Co rapidly dissolved into the solution compared to the other elements, as depicted in Figure 3. Even though Al and Co quantities were less, their rapid diffusion could be attributed to their relatively low melting temperatures.

Other elements such as Cr and Fe dissolved into solution as the milling time increased. Cr and Fe exhibit a BCC crystal structure and therefore may accommodate other elements without much expansion [114,126]. Additionally, Cr has the highest melting temperature in the HEA system and thus diffuses the slowest during the MA process. The MA process is known to include repeated welding and fracturing, leading to crystallite refinement. Since HEAs are baseless systems composed of elements with different atomic sizes, the size mismatch effect occurs during the MA process. Increasing milling time during the MA process leads to an increase in dislocation density and lattice strain caused by the severe plastic deformation occurring, and the size mismatch effect and increase in the grain boundary fraction owing to the crystallite refinement [127].

Vaidya et al. [81] studied the effect of the elemental addition sequence on the phase evolution of the nanocrystalline AlCoCrFeNi HEA system using the MA processing technique. Three different classes of binaries were selected as initial starting phases, while other constituent elements were added stepwise in varying sequences until quinary systems were formed. The binaries included B2 (AlNi, AlCo and AlFe), BCC (FeCr), and FCC (CoNi and FeNi) phases. For example, in AlCo starting as a binary lattice, the more the FCC phase from the dual BCC + FCC phase expands, the more Al dissolves into the solid solution. The addition of Ni to AlCo (B2) favors an FCC structure instead of a stabilizing B2 structure. This indicates a high tendency of an FCC solid solution of Ni (Co) forming than an intermetallic AlNiCo phase. The formation of AlCoNiFeCr from AlCoNi due to the addition of Fe and Cr destabilizes the FCC phase. As a result, the BCC phase evolves from FCC to form BCC (major) + FCC as the final microstructure. Praveen et al. [128] also reported a similar phase during MA of CoCrFeNi alloy. There were variations in the amount of BCC and FCC present at the end of each sequence in the CoCrFeNi HEA fabricated. The resulting microstructure of AlCoNiFeCr HEA, BCC + FCC, remained consistent with a wide range of sequences despite variation in both alloying sequence and milling duration. This is contrary to the single-phase BCC formation reported when FeNiCoCrAl, FeCrAlNiCo, AlNiCoFeCr alloy sequences from three different binaries were processed using arc-melting [129,130]. In addition, the base alloy sequence AlCoCrFeNi exhibited a single-phase BCC structure when fabricated using arc-melting [129].

It is noteworthy that the alloy sequences starting with the BCC phase showed a larger amount of FCC phase fractions at the end of milling, and this phase becomes stable when nanocrystalline Ni is added. Therefore, it can be inferred that the FCC phase is promoted by Ni and Co addition, unless when Co is added immediately after Al owing to Co’s strong affinity to Al. The BCC phase is stabilized by the addition of Al during sequential alloying irrespective of the position at which it is added [20,120]. Cr favors BCC phase formation, while Fe has the least influence on phase formation due to a less negative ΔH_avg_ and the lowest atomic radius. It can be deduced that the initial structure of the binary alloy, the addition of individual elements, and the order of mixing constituents are some of the factors that influence the phase evolution during sequential alloying in the MA route.

**Figure 4:**
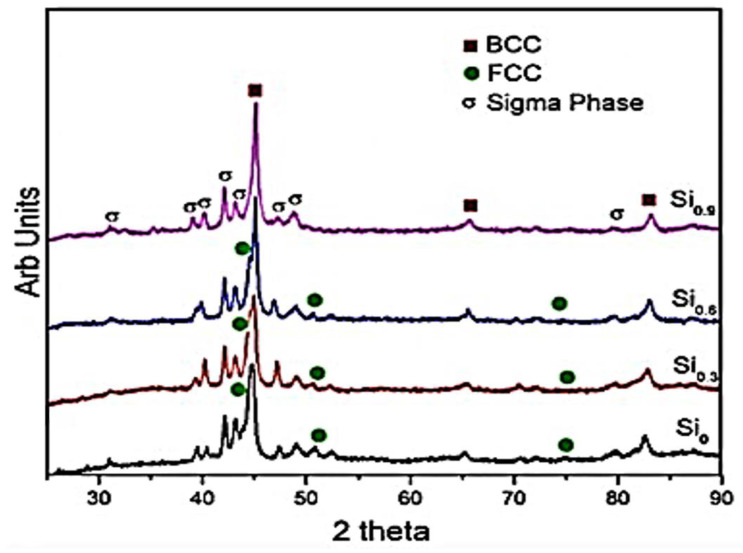
XRD pattern of AlCoCrFeNiSi_x_ after SPS, where x = 0, 0.3, 0.6, and 0.9 [115].

Furthermore, the formation of the supersaturated BCC solid solution in the FeNiCrCo_0.3_Al_0.7_ HEA is attributed to the energy stored in the grain boundaries, high entropy of mixing, and solid solubility extension [114,131]. Partial BCC phases in the FeNiCrCo_0.3_Al_0.7_ HEA after MA evolved to a more stable FCC phase during the SPS process at 1000 °C. The annihilation of defects introduced into the structure owing to the high-energy ball milling during the MA process leads to reordering at high temperatures. As a result, defects introduced by severe plastic deformation during MA are nearly annihilated after consolidation by SPS, which can also contribute to phase evolution during SPS. Ji et al. [112] obtained a BCC solid solution for CoCrFeNiAl HEA after milling for 60 h. From the study, the BCC solid solution formed during the first 30 h and was only refined as milling progressed to 60 h. After SPS consolidation at 900 °C, BCC and FCC phases formed. Kumar et al. [115] also achieved a BCC supersaturated solid solution by MA of AlCoCrFeNiSi_x_ for 20 h. The studied alloy was quite similar to that of Ji et al. However, a BCC solid solution was attained after a shorter milling duration (20 h) compared to the 60 h done by Ji et al. [112]. This goes to show that milling speed also influences the phases formed during the MA process. Kumar et al. [115] used a milling speed of 300 rpm, while Ji et al. [112] used a speed of 250 rpm. Major phases composed of BCC, FCC, and sigma were obtained in the AlCoCrFeNiSi_x_ (x = 0.3, 0.6, and 0.9) fabricated by MA after SPS consolidation at 1000 °C. However, only BCC and sigma phases were observed in AlCCrFeNiSi_0.9_. Figure 4 shows the XRD pattern of AlCoCrFeNiSi_x_ after SPS. Si is known to be a BCC former and stabilizer. Si, having the ability to occupy another element spot in the grain boundary, shows that more atoms will be replaced with an increase in the Si content [115]. This, thereby, introduced lattice distortion and lattice strain in the system, while it destabilized the FCC phase.

## 4. Microstructural Evolution of HEAs Fabricated by Additive Manufacturing (AM)

**Figure 5:**
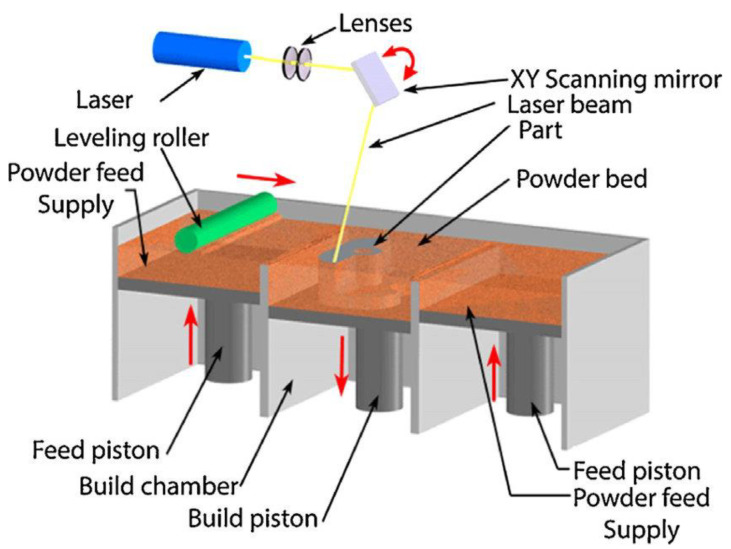
Schematic representation of additive manufacturing techniques [133].

AM has become a mainstream manufacturing process because of its flexible design optimization and processing advantages. The production of customized parts and the ability to control the microstructure in a specific site are possible in this processing route. The higher heating and cooling rates associated with AM promote chemical homogeneity in alloys by restricting diffusion to avoid undesired multiple phase transformations during cooling [132]. Solidification mainly takes place along the building direction and is predominantly epitaxial. The successive building process in thin layers by local heat input characterizes the microstructures as a result of rapid and directional solidification. Factors such as powder flowability, laser power, powder thickness and shape, scan spacing, and volumetric energy density (VED) all play an important role in determining the resulting microstructure in AM technology. Figure 5 below shows the schematic representation of additive manufacturing techniques.

Table 3 presents some HEAs fabricated using the AM route. The AlCrFeCoNi HEA system has also been synthesized by Kuwabara et al. [134] and Fujieda et al. [135] using the selective electron beam melting (SEBM) AM technique. The microstructure of the SEBM HEA exhibited a BCC and a B2 phase, same as that reported when processed through the melting and casting route, despite the rapid solidification of the SEBM process [16,28]. In addition to the BCC microstructure, an FCC phase was observed at the bottom of the SEBM-fabricated HEA. The precipitation of the FCC phase could have resulted from the BCC or B2 phase in a lower temperature range during building. Moreover, the phase evolution could have also occurred during the preheating process, which is associated with the SEBM AM technology. The coexistence of BCC and FCC phases in AlCrFeCoNi was confirmed when Ji et al. [112] fabricated the same HEA using the powder metallurgy (MA + SPS) approach.

Jung et al. [136] fabricated an AlCrFeCoMnNi HEA using the laser powder bed fusion (LPBF) process. Again, the microstructure revealed B2 (Ni-Al-rich) and BCC (Fe-Cr-rich) solid solutions. This is in contrast with the formation of BCC and FCC phases achieved in the same alloy when synthesized using either powder metallurgy or the melting and casting process [60,118]. The microstructure formation of the AlCrFeCoMnNi HEA fabricated by LPBF was attributed to (1) liquid-phase spinodal decomposition from an undercooled melting, (2) the cubic nature of the HEA and the highly textured microstructure associated with LPBF, and (3) properties of alloying elements, such as Mn, Al, and Zn volatility at high temperature. Depletion of such elements at the melt pool surface leads to a reduction in the distribution of elemental composition across different layers. For instance, depletion of Al in the AlCrFeCoMnNi HEA results in variation in the Al content across built layers, which leads to a variation in the phase transition temperatures and the phase composition.

Fine BCC grains were found distributed at the grain boundaries of the FCC matrix when Gao et al. [137] fabricated a CoCrFeMnNi HEA using laser 3D printing technology. This is in contrast with the single-phase FCC achieved by Kucza et al. [14] Tsai, et al. [92], Pickering et al. [11], and Yao et al. [46], who that processed the same HEA using different forms of melting and casting routes. Joo et al. [138] also confirmed the presence of single-phase FCC in CoCrFeMnNi when fabricated using the powder metallurgy technique. FCC (major) matrix grain boundaries were partially wetted by a BCC solid second phase in the printed HEA. Grain boundary wetting phase transformation is known to be responsible for the morphology of the second solid phase in the grain boundaries of the first solid phase. Al_0.3_CoCrFeNi exhibited single-phase FCC when processed through LPBF [139]. A similar alloy was fabricated using the melting and casting route, yet it showed the same microstructure [47]. However, rapid solidification and anisotropic heat removal associated with LPBF generate fine columnar grains in the HEA. This factor seems to not influence the Al_0.3_CoCrFeNi microstructure in this case. There is consistency in the microstructure of AlCoCrFeNiTi_0.5_ when fabricated using both laser-engineered net shaping (LENS) [140] as well as the melting and casting route [141]. The microstructure exhibited both disordered and ordered BCC phases. However, Shaofeng et al. [116] reported a mixture of BCC and FCC in the same alloy when fabricated using MA + SPS. A laser-engineered net-shaping-processed AlCoCrFeNiTi_0.5_ HEA contains a fully equiaxed grain microstructure rather than a columnar microstructure mostly associated with alloys fabricated by AM [142,143,144]. Hence, an AlCoCrFeNiTi_0.5_ HEA defies the norms in this regard. Luo et al. [145] reported a BCC solid solution in a selective laser-melting-processed AlCrCuFeNi HEA. However, Jinhong et al. [73] and Anmin et al. [74] reported the presence of FCC and BCC in the same as-cast alloy. The presence of FCC and BCC phases was also confirmed in the same HEA using the powder metallurgy approach [120]. The FCC phase formation might have been inhibited as a result of rapid cooling during the selective laser melting fabrication process [73]. In this fabrication process, the high lattice distortion and elastic strain induced in the HEA is a result of the rapid cooling.

## 5. Mechanical Properties

The strengthening effect in a traditional solid solution is achieved as a result of the mismatch between solute and solvent causing a strain field in the alloy [168]. Some scholars believe it is difficult to differentiate between solvent and solute in HEAs due to their equal or near-equal chemical compositions [169]. Therefore, it is a challenge to evaluate the strengthening effect by applying traditional solid-solution-strengthening mechanisms to HEAs. Researchers have come up with several mechanistic theories to predict the plastic yield strength, especially in FCC HEAs. Labusch-type models of Varvenne, Luque, and Curtin are some of these parameter-free theories [170,171]. They predict the plastic yield strength of FCC HEAs as a function of temperature, composition, and strain. The successful application of conventional or traditional strengthening methods on HEAs results in a reduction in toughness [172]. The strengthening mechanisms in HEAs can be summarized as solid solution hardening (σ_s_), precipitation hardening (σ_d_), grain boundary strengthening (σ_g_), and dislocation hardening (σ_p_). Hence, the yield strength of an HEA can be expressed as the summation of every individual contribution [170].

σ_0.2_ = σ_0_ + Δσ_s_ + Δσ_d_ + Δσ_g_ + Δσ_p_ (1)

where σ_0_ is the yield strength of the alloy, which is the intrinsic strength, or lattice friction strength.

### 5.1. Solid Solution Hardening

Defining or evaluating the contribution of solid-state strengthening in HEAs remains a challenge. This implies that the mechanism of hardening is not yet well understood. Any attempt to apply traditional solid-solution-hardening theories to HEAs proves abortive. For instance, the yield strength value realized in the FeCoNiCr solvent matrix containing the Ti + Al solute is too small to account for the strength difference in the HEA [173]. Hence, it can be assumed that solid solution hardening is not the dominant mechanism in this process. According to Senkov et al. [39], the high hardness (5.25 GPa) attained in the high-entropy BCC-phase WNbMoTaV alloy cannot be attributed to solid solution hardening. Some researchers believe that solid solution hardening is the main cause of the exceptional mechanical properties of HEAs.

### 5.2. Precipitation Hardening

Precipitation strengthening is another way by which HEAs have been strengthened, and modes of strengthening are categorized based on different interaction mechanisms [174]. For instance, (1) stacking-fault strengthening, (2) modulus strengthening, and (3) chemical strengthening are grouped among modes of precipitation hardening when dislocation cuts through particles [175,176]. Less attention is given to the above-mentioned modes of precipitation strengthening since they contribute much less to the yield strength in HEAs. Other modes of precipitation hardening are coherency strengthening and ordering strengthening [177]. Other modes of precipitation hardening have also been used to strengthen HEAs, especially FCC HEAs. Here, fine precipitates are expected to generate hardening either through a particle-shearing mechanism or through a dislocation bypass mechanism (Orowan type). The shearing mechanism occurs when precipitates are sufficiently small and coherent [177]. However, in the Orowan mechanism, the radius of particles is incoherent with the matrix or the particles are not easy to cut through; hence, hardening exceeds a critical value [178]. Ashby-Orowan predicted the yield strength caused by the Orowan mechanism through the relation below [179]:

Δσ_orowan_ = 0.538 G*b*ƒ^½^/r × ln(r/2*b*) (2)

In some HEAs, yield strength has been successfully enhanced by improving the volume fraction of coherent $\gamma^{\prime }$ phase through an increase in the Al and Ti concentrations [168,180,181]. In superalloys, the $\gamma^{\prime \prime }$ phase as a precipitate has been demonstrated to have a better strengthening effect than $\gamma^{\prime }$ due to its higher anti-phase boundary energy and higher lattice misfit [182]. An excellent combination of yield strength (954 MPa) and ductility (27%) was realized in an Ni_2_CoCrFeNb_0.15_ HEA by enhancing its yield strength by 670 MPa using the $\gamma^{\prime \prime }$ precipitate [177]. For the $\gamma^{\prime \prime }$ phase, yield strength has mostly been reported as a result of both coherency and ordering mode of the strengthening mechanism. Equations for coherency and ordering strengthening for the $\gamma^{\prime \prime }$ phase can be found below [183]:

Δσ_coherency_ = 1.7MG|ε|^3/2^ × [h^2^ƒ (1 − β)/2*bR*]^½^ (3)

(4) $${\Delta \sigma \gamma^{\prime \prime }}_{\mathrm{ordering}}=M(\gamma \mathrm{APB}/2b)\{[4\gamma \mathrm{APB}f/\pi T\;\times \;(\surd 6\mathrm{Rh}/3{)}^{½}{]}^{½}-\beta f\}$$

where G is shear modulus, ƒ is the volume fraction of precipitates, ε is the tetragonal lattice of misfit, *b* is the magnitude of Burger’s vector, h is the half-thickness of the particles, R is the real diameter of the particles, γ APB is the antiphase boundary energy of the $\gamma^{\prime \prime }$ phase, β is a constant and equal to ⅓ when all three variants are observed, *T* is the line tension, and M is Taylor factor (3.06 for an FCC polycrystalline matrix).

However, aside from difficulties associated with designing $\gamma^{\prime \prime }$ phase(precipitates) for multiphased HEAs, it is also a challenge to precisely measure or control the chemical composition of precipitates. Eißmann et al. [184] demonstrated how the precipitation hardening method was used to increase the Cantor alloy hardness. Precipitation hardening was used to reach the maximum hardness of 353 HV in Ti-6-750-10, exceeding the cantor alloy by a factor of 2 [184]. He et al. [168] attributed the strength increment of about 326.7 MPa achieved in FeCoNiCr HEA to precipitation hardening.

### 5.3. Grain Boundary Strengthening

The smaller the grain size, the higher the volume fraction of grain boundaries, which hinders dislocation motion and thereby improves the strength of HEAs. The relationship between yield strength and grain size is well described by the Hall–Petch equation [185,186]:

σ_y_ = σ_0_ + k_y_/d^½^ (5)

where σ_y_ is the yield stress, σ_0_ is the lattice friction stress, d is the average grain diameter, and k_y_ is the strengthening coefficient. From Equation (4), an increase in yield strength as a result of grain size difference (Δσ_G_) can be expressed as

Δσ_G_ = k_y_ × (d_p_^−½^−d_A_^−½^) (6)

where d_p_ represents the grain size of the thermomechanically processed materials.

The value of the yield strength increase caused by the grain size difference obtained in FeCoNiCrMn is too small to account for the total strength increase in the HEA [187]. The hardness of 580 HV realized in CoCrFeNi was attributed to precipitation strengthening and grain boundary strengthening [23]. Liu et al. [188] also attributed the high tensile strength of 712.5 MPa, as well as the high elongation of 56%, to grain boundary strengthening in the same alloy. Ganji et al. [189] showed that grain boundary strengthening contributes about 85% of flow stress in AlCoCrCuFeNi HEA. Strain hardening and grain boundary strengthening were reportedly responsible for the hardness of 8.13 GPa and an elastic modulus of 172 GPa achieved in a dual-phase (FCC + BCC) AlCoCrCuFeNi HEA [189].

### 5.4. Dislocation Hardening

Dislocation hardening is caused by interaction between solute atoms of different sizes and properties, resulting in an elastically disorganized crystal lattice. This brings about the formation of a local elastic stress field for an increase in strength to take place. An increase in strength is achieved as a result of interaction between mobile dislocations, as they hinder their own movement. Hence, a higher dislocation density leads to a higher yield strength. The Bailey–Hirsch equation can be applied to describe the relationship [190]:

Δσ_D_ = M α *Gbρ*^½^ (7)

where *ρ* stands for the dislocation density, *b* is the burger vector, and α is a constant (e.g., 0.2 for FCC metals).

He et al. [168] demonstrated that a good balance between yield strength and ductility can be achieved in FCC HEAs through good use of grain boundary hardening, dislocation hardening, and precipitation hardening. Studies have also shown that phase transformation can be triggered by small interstitial solutes such as carbon or boron during solid solution strengthening. Research has also proved that boride and carbide compounds precipitate in some HEAs such as Al_0.5_CoCrCuFeNiB_x_ and Al_0.3_CoCrFeNiC_0.1_ when fabricated using arc-melting [191]. However, the presence of an energy barrier stabilizes the interstitial solid solution. Improved mechanical properties were achieved on an FeCoNiCrCuTiMoAlSiB_0.5_ HEA as a result of an interstitial solute and laser rapid solidification [191]. The presence of an interstitial solute and other factors co-triggered the nucleation of the martensitic phase, which contributed to improved properties in the FeCoNiCrCuTiMoAlSiB_0.5_ HEA. Table 4 below summarizes different strengthening mechanisms used in improving the mechanical properties of HEAs processed by various fabrication methods and their resulting microstructures as well as the respective phase(s) achieved.

It can be deduced that the synthesis route has little or no influence on the HEA microstructure. This was the case when the noble cantor alloy CoCrFeNiMn maintained an FCC phase when processed through melting and casting [11,46] as well as MA + SPS [192], but the FCC + BCC phase was reported when fabricated via AM technology [137,146,147]. AlCoCrCuFeNi was also fabricated using the melting and casting route [64] as well as the MA + SPS route [189], both resulting in an FCC + BCC phase. The BCC phase was achieved in the same HEA when fabricated via the AM route [154]. Furthermore, it is difficult to evaluate the effect of solid-solution-strengthening mechanisms on HEAs’ mechanical properties with respect to the synthesis route and resulting microstructure. This is evident when the hardness achieved in an FCC + BCC AlCoCrCuFeNi HEA processed through the melting and casting route and MA + SPS is recorded as 515.5 HV (5.056 GPa) [64] and 8.13 GPa [189], respectively, while a Ni_1.5_Co_1.5_CrFeTi_0.5_ FCC phase HEA processed through the same melting and casting route and MA + SPS reportedly had a hardness of 515 HV [48] and 442 HV0.3 [119], respectively. Hence, greater hardness is achieved in an AlCoCrCuFeNi HEA processed through MA + SPS than when synthesized using the melting and casting method. However, the reverse is the case when a Ni_1.5_Co_1.5_CrFeTi_0.5_ HEA is processed through the same set of fabrication methods despite the two HEAs being strengthened by solid solution strengthening and grain boundary strengthening.

## 6. Concluding Remarks

The quest to achieve specific mechanical properties in HEA systems to meet demands in engineering applications has attracted the attention of researchers over the past decade. This calls for a need to review the processing–structure relationship in HEAs to help in predicting resulting properties. This paper reviewed the relationship between the common processing routes (melting and casting, PM, and AM) and possible structure(s) formed, as well as factors that may influence the properties. The content of this paper can be summarized as follows.

During the melting and casting process, the phase transformation of HEAs occurs during solidification (cooling). During solidification, phase evolution depends on the collective mobility or distribution of constituent elements making up the alloy. However, the rate of cooling, differences in the local atomic arrangement, and the varying elemental diffusivity can influence the solid phase that is first to form and the microstructure of the alloys. HEAs fabricated using the melting and casting route usually show dendritic microstructures with interdendritic segregations. Thus, melting and casting techniques with faster cooling rates favor the formation of a more dominant single phase and limit the precipitation of secondary phases. From another perspective, HEA phase formation during fabrication via the melting and casting route is dependent more on binary constituent pairs than individual constituent elements.

The energy involved during the MA process generates heat and influences the phases that form. Severe plastic deformation generated during the MA process leads to an increase in dislocation density, and lattice strain is introduced into the system. The milling duration/speed, ball-to-powder ratio (BPR), and grinding media contribute to the phase evolution in the powders. The initial structure of binary alloy pairs, sequential addition of individual elements, and order of mixing constituents are some of the factors that also influence the phase evolution. Lattice distortion and sluggish diffusion of elements during the SPS process do influence phase evolution in HEAs. It can also be deduced that the microstructure and phases achieved in the final sintered HEAs can be significantly influenced by the processing parameters used during MA and SPS processes.

In AM technology, some elements such as Mn, Al, and Zn mainly deplete at the main melt pool surface at high temperatures due to their high vapor pressure. This leads to a reduction in the distribution of elemental composition across different layers. Variation in the composition across layers results in compositional inhomogeneities in HEAs. For sensitive HEAs, changes in compositions have been seen to have a strong impact on the as-built microstructure. In particular, variation in the Al content across built layers leads to variation in phase transition temperatures and the phase composition. In addition, solidification mainly takes place along the building direction and is predominantly epitaxial. The successive building process in thin layers by local heat input does characterize the microstructures as a result of rapid and directional solidification. Factors such as powder flowability, laser power, powder thickness and shape, scan spacing, and volumetric energy density (VED) all play an important role in determining the resulting microstructure in AM technology.

## 7. Recommendations for Future Studies

Large differences in the melting temperatures of the constituent elements due to compositional complexity result in elemental segregation, dendritic structure, and residual stress in HEAs fabricated using the melting and casting route. These have a direct impact on the properties of the materials produced to date and could explain some of the discrepancies found within the same-composition alloys produced through the melting and casting route. To address these discrepancies, the rate of cooling, differences in the local atomic arrangement, and the varying elemental diffusivity must be taken into consideration in future studies. Faster cooling routes such as suction casting, injection casting, melt spinning, or splat cooling suppress the precipitation of the secondary phase and thereby form a predominantly stable single-phase structure. Hence, induction remelting can reduce microsegregation, reduce the inhomogeneity challenge, and refine the grain size.

Most of the studies on HEAs fabricated by MA are focused on varying the milling duration in achieving a homogeneous solid solution of the elements. However, since the parameters of the MA process are not independent of each other, it is imperative to know that other parameters such as milling speed, the BPR, grinding media, and the milling environment are given some attention in future studies. These other parameters also significantly influence the heat generated during milling and the diffusion of elements in the solid solution process. A lower sintering temperature (depends on the melting temperatures of constituent elements) should also be considered.

There is no adequate information to better understand how, where, and why voids and porosity were formed in most AM-fabricated materials. More attention is needed in this area as controlling their distribution or avoiding them is crucial and requires a better understanding; hence, these defects are undesirable in certain engineering applications. As with all AM technologies, there remains an ongoing quest to reduce cost, improve speed, and improve robustness. However, process validation remains an issue as the quality of the printed material is still in check. Therefore, there is a need for the development and standardization of economically viable and printable materials for engineering applications in the AM fabrication technique spectrum to complement its processing advantages. Urgent attention is needed in developing computer-aided design tools and predictive models of both the printing process and the post-printing material properties in future studies.

Although Li et al. [198], Borkar et al. [199], Welk et al. [200], and more researchers have used the combinatorial approach in processing multicomponent alloys, more attention is still needed on this method owing to the possibility of exploring composition space. This will allow the measurement of a variety of properties across the composition array. Thus, observations suggest that proper selection of the chemical composition and an appropriate processing route combined with appropriate thermomechanical treatment may offer an opportunity to manipulate the strengthening mechanism to enhance HEAs’ mechanical properties. An optimal composition with required properties could be more efficient. Their microstructure and properties can be effectively examined. For instance, the lattice distortion that simultaneously emanates from the presence of solutes needs to be measured. Hence, the effect caused by the distortion on dislocation movement is not clear and needs to be understood. In addition, diffusion data and models are critical to understanding the microstructural evolution of HEAs for easy prediction of practical features such as grain growth, growth of strengthening phases, and nucleation. Therefore, more research with modeling and simulations is required, in addition to computational tools and integrated computational material engineering available.

## Figures and Tables

**Table 1:** Phase evolution of HEAs fabricated using the melting and casting route.

HEA Composition	Processing Method	Observed Phase(s)	Microstructures and Comments	Reference
AlCoCrFeNi	Arc-melting	BCC	A dendritic structure is included.	[26,28]
AlTiVCr	Arc-melting	Single phase consisting of a B2 phase and a disordered BCC phase	The B2 phase is more stable than the disordered BCC phase.	[33]
AlCoFeNiTi	Arc-melting	BCC	A dendritic structure is included.	[34]
TiVZrNbHf	Arc-melting	Single-phase BCC		[35]
AlCrFeNiMo_0.2_	Vacuum Induction	BCC and B2 structure	The BCC phase is FeCrMo-rich, while the B2 phase is a NiAl-rich intermetallic compound.	[36]
NbCrMoTiAl_0.5_	Arc-melting	Simple BCC	Mo segregates to the dendritic region.	[37]
NbCrMoTiVAl_0.5_Si_0.3_			Cr, Ti, Al, and Si segregate to the interdendritic regions.	
Al_x_CoFeNiSi (x > 0.3)	Arc-melting	BCC		[38]
MoNbTaVW	Arc-melting	Single BCC	Dendritic and interdendritic regions are present due to constitutional segregation during solidification.	[39,40]
Al_x_CrFeMnNi_0.5_	Arc-melting	BCC		[41]
(x = 0.8–1.2)				
Nb_25_Mo_25_Ta_25_W_25_	Arc-melting	BCC phase	There is no dendritic segregation.	[15]
Fe_36_Mn_21_Cr_18_Ni_15_Al_10_	Arc-melting	Dual-phase 2 BCCs/B2	The matrix phase (BCC) is rich in Fe and Cr.	[42]
			The B2 phase is rich in Ni and Al.	
CoCrCuFeNi	Arc-melting	FCC	The interface morphology would grow in planar, cellular, and dendrite if the solidification rate is increased.	[18,43]
CoCrFeNiV_0.5_C_x_	Arc-melting	FCC	A large number of M_7_C_3_-type interstitial carbides are formed at an annealing temperature of 700 °C and above.	[44]
(x = 0.01, 0.02, 0.03, and 0.04)				
Fe_40_Mn_40_Co_10_Cr_10_	Vacuum induction	FCC		[45]
CrMnFeCoNi	Arc-melting, Vacuum Induction	FCC	Precipitates of M_23_C_6_ and the *σ* phase exist following prolonged exposure at 700 °C.	[11,46]
Al_x_CoCrFeNi	Arc-melting	FCC	The FCC phase is transformed to the BCC phase with the presence of a transition duplex FCC/BCC region as Al increases.	[47]
(x = 0–0.65)				
CoCrFeNiTi_0.3_	Arc-melting	FCC	A crystalline structure is present consisting of a mixture of a (Ni, Ti)-rich R phase and a (Cr, Fe)-rich σ phase within the FCC matrix.	[48]
Al_0.5_CoCrCu_0.5_FeNi	Arc-melting	FCC	The BCC phase will evolve from the FCC phase with an increase in the Al content.	[49]
			FCC + BCC duplex phases will evolve at Al (0.5–1.5).	
CoCrFeNiNb_0.25_	Arc-melting	FCC	Lath-shaped FCC precipitates + nano-basket-weave microstructures are randomly distributed in the proeutectic FCC phase.	[50]
Al_x_CoCrFeNiTi_y_	Arc-melting	FCC	The Al and Ti content strongly affects the phase and microstructure.	[51]
Co_1.5_CrFeNi_1.5_Ti_0.5_Mo_x_	Arc-melting	FCC	An interdendritic phase, (Ni, Ti)-rich phase and dendritic (Fe, Cr)-rich phase are present when x = 0, 0.1.	[52]
(x = 0, 0.1)				
Mn_22.3_Fe_22.2_Ni_22.2_Ge_16.65_Si_16.65_	Arc-melting	FCC	Magneto-structural first-order phase transition is exhibited.	[53]
AlCrFeMnNi	Arc-melting	BCC (B2) + FCC	The BCC phase is interdendritic and rich in Al + Ni.	[54]
Ni_30_Co_30_Cr_10_Fe_10_Al_18_W_2_	Arc-melting	FCC + BCC	Fine, regular, lamellar eutectic + coarse irregular eutectic hierarchical microstructures are present.	[55]
Al_0.5_CrFeMnNi_0.5_	Arc-melting	FCC + BCC	A dendritic region (higher Al and Cr) and an interdendritic region are present.	[56]
			Precipitates (AlNi B2 compound) are present.	
Al*_x_*CoCrFeNi	Arc-melting	FCC + BCC	An AlNi-rich precipitate is formed.	[57]
(x = 0.45–0.85)				
Cr_2_Cu_2_FeNi_2_Mn_2_	Arc-melting	FCC + BCC	A dendritic and interdendritic phase is present.	[58,59]
Cr_2_Cu_2_NiMn_2_				
CrCu_2_Fe_2_NiMn			Cu, Mn, Cr, and Fe are segregated in dendritic/interdendritic regions, while Ni is homogeneously distributed in the alloy.	
Cr_2_CuFe_2_NiMn				
Al*_x_*(CoCrFeMnNi)_100−*x*_	Arc-melting	FCC + BCC	An increase in Al turns the dendritic structure to a lamella-like structure, hence the transit from the FCC to the BCC phase.	[60]
CoCrFeMnNiZr_x_ (x = 0–0.3)	Arc-melting	FCC + BCC	Dendritic and interdendritic regions are present.	[61]
			The interdendritic region increases with an increase in the Zr content.	
AlCoCrCu_x_NiTi	Arc-melting	FCC + BCC	Dendritic (contains compound impurities) and chrysanthemum-shape dendrites are present.	[62]
(x = 0.5–0.8)			Cu segregates in the interdendritic region.	
CoCu_y_FeNiTi_x_	Arc-melting	2 FCCs + BCC	FCC 1 is Cu rich, and FCC 2 is Co rich (x = 1/3, 3/7, and 3/5).	[63]
			The BCC phase is β Ti rich (x = 3/5).	
CoCrFeNiCuAl	Arc-melting	FCC + BCC	A cast-dendritic morphology is present.	[64,65]
			The BCC phase is an ordered one.	
			of 2 FCC phases are present.	
Fe_50-X_Mn_30_Co_10_Cr_10_B_X_	Arc-melting	FCC + BCC	The addition of boron promotes the formation of M_2_B-type borides (M = Cr, Fe).	[66]
(x = 0, 0.3, 0.6, 1.7 wt%)				
AlCrCuFeMnNi	Vacuum Induction	2 BCCs (B2 + A2) + FCC	The 2BCC phase is formed by spinodal decomposition, i.e., B2 (NiAl dendrite matrix) and A2 (Cr-Fe rich) embedded precipitate.	[67]
Al_0.5_CoCrFeNi	Arc-melting, Vacuum Induction	FCC + BCC crystalline structures	The presence of the Al-Ni-rich phase decreases as the aging temperature increases and, hence, leads to an increase in the amount of Al-(Ni, Co, Cr, Fe).	[68,69]
NbMoTaTi–(W, V)	Arc-melting	BCC + HCP—with W inclusion	The HEA with “V” shows a dendritic/cellular microstructure rich in Ti and V.	[70]
		BCC—with V inclusion	The HEA with “W” forms a Ti-rich HCP phase.	
Al_0.5_CrCuNiV	Arc-melting	FCC + 2 BCCs + B2	A dendrite rich in Cr and V is present.	[71]
			The incorporation of Cu into the 2-BBC phase differentiates it from the B2 phase.	
AlCoCrFeNi_2.1_	Vacuum Induction	Dual-phase FCC + BCC (B2)	-	[72]
AlCrCuFeNi	Arc-melting	FCC + BCC	The content of Ni has a significant effect on the HEA microstructure.	[73,74]
(0.6 *≤* x ≤ 1.4)				

**Table 2:** Phase evolution of HEAs after MA and SPS.

HEA Alloy	MA Parameters	SPS Parameters	Phase Evolution		Reference
			MA	After SPS	
FeNiCrCo_0.3_Al_0.7_	S = 300 rpm	ST = 600 °C (4 min)	BCC	BCC + FCC	[114]
	BPR = 10:1	HR = 75 °C min^−1^			
	D = 45 h	ST = 600 to 1000 °C (at HR = 50 °C min^−1^ in 4 min)			
	GM = stainless steel vial, tungsten carbide balls	(1000 °C in 8 min),			
		P = 30 MPa			
CoCrFeNiAl	S = 250 rpm	ST = 900 °C (10 min)	BCC after first 30 h of MA	BCC + FCC	[112]
	BPR = 15:1				
	D = 60 h	P = 50 MPa			
	Annealed from 500–1000 °C for 1 h	Cooled to 600 °C in 5 min			
	GM = stainless steel vial and balls				
AlCoCrFeNiSi_x_ (x = 0.3, 0.6, and 0.9)	S = 300 rpm	ST = 570–800 °C	BCC	BCC + FCC + sigma phase	[115]
		HR = 100 °C min^−1^			
	BPR = 10:1	ST = 800 °C–1000 °C			
	D = 20 h	HR = 50 °C min^−1^			
	GM = tungsten carbide vial	(1000 °C in 5 min),			
		P = 60 MPa			
Al_0.4_FeCrCo_1.5_NiTi_0.3_	S = 300 rpm	ST = 1000 °C (10 min)	BCC + FCC	FCC (major) + BCC (minor)	[116]
	BPR = 10:1				
	D = 50 h (dry) + 5 h (wet)	P = 30 MPa			
Al_0.5_CrFeNiCo_0.3_C_0.2_	S = 300 rpm	ST = 600 °C (4 min)	BCC + FCC within first 38 h MA	FCC (major) + BCC	[117]
	BPR = 10:1	ST = 600–900 °C			
		HR = 75 °C min^−1^			
	D = 38 h dry + 4 h wet (42 h)	ST = 900–1000 °C			
		HR = 50 °C min^−1^			
	GM = stainless steel vial, tungsten carbide balls	(1000 °C in 8 min)			
		P = 30 MPa			
CoCrFeNiMnAl	S = 250 rpm	ST = 800 °C (10 min)	BCC	BCC + FCC	[118]
	BPR = 15:1				
	D = 60 h	P = 50 MPa			
	Annealed from 500–1000 °C				
	GM = stainless steel vial and balls, N-heptane PCA				
Ni_1.5_Co_1.5_CrFeTi_0.5_	S = 250 rpm	ST = 1000 °C at HR = 100 °C min^−1^	BCC + 2 FCCs	FCC + oxide	[119]
	BPR = 10:1	ST = 1000–1100 °C at HR = 50 °C min^−1^			
	D = 30 h dry + 2 h wet (toluene) (32 h)	ST = 1100–1150 °C at HR = 20 °C min^−1^			
	GM = hardened tool steel vial and hardened balls	(1150 °C in 20 min)			
		P = 30 MPa			
AlCuNiFeCr	S = 580 rpm	ST = 700, 800, and 900 °C (15 min)	BCC	B2 + FCC + (Fe,Cr)_23_C_6_ after SPS	[120]
	BPR = 1:10				
	D = 5 h	P = 150 MPa			
	GM = hardened ShH-15 steel, gasoline medium				
Nb_25_Mo_25_Ta_25_W_25_	S = 400 rpm	ST = 1600 °C (8 min)	BCC	BCC	[121]
	BPR = 15:1				
Ti_8_Nb_23_Mo_23_Ta_23_W_23_	D = 60 h	P = 35 MPa			
	GM = tungsten carbide vials, acetone PCA				
CoNiFeAlTi	S = 300 rpm	ST = 1000 °C (8 min)	BCC + FCC	BCC (B2) + FCC + Al_3_Ti intermetallics after SPS	[122]
	BPR = 10:1				
	D = 4 h wet + 45 h dry (49 h)	HR = 90 °C min^−1^			
	GM = stainless steel vials and tungsten carbide balls, no PCA	P = 30 MPa			
Al_0.3_CoCrFeMnNi	S = 200 rpm	ST = 800, 900, and 1000 °C (10 min),	FCC	BCC (B2) after SPS	[123]
	BPR = 15:1	HR = 100 °C min^−1^			
	D = 36 h				
	GM = stainless steel vials and balls, N-heptane PCA	P = 50 MPa			
(CuCrFeTiZn)_100-x_Pb_x_ (x = 0, 5, 10, and 20)	S = 200 rpm	ST = 800, 900, and 1000 °C	Fe-Cr (BCC) + Cu-Zn (FCC)	Fe-Cr (BCC) + Cu-Zn (FCC)	[124]
	BPR = 20:1				
	D = 44 h	HR = 150 °C min^−1^			
	GM = tungsten carbide vials and balls	P = 50 MPa			

Legend: S, milling speed; BPR, ball-to-powder ratio; D, milling duration; ST, sintering temperature; HR, heating rate; P, compression pressure; GM, grinding media.

**Table 3:** Phase evolution of HEAs fabricated using different additive manufacturing routes.

HEA Composition	Processing Method	Observed Phase(s)	Microstructures and Comments	Reference
CoCrFeMnNi	Laser 3D printing	FCC (major) + BCC	An equiaxed-to-columnar transition structure was discovered in the melt pool.	[137]
CoCrFeMnNi	Laser powder bed fusion (LPBF)	FCC + σ phase	Nanotwins were present in the printed sample.	[146]
			Mn segregates at the boundary of the weld pool due to its volatility.	
CoCrFeMnNi	Laser directed energy deposition	FCC solid solution	No phase transformation occurred	[147]
			Lattice strain and grain refinement occurred.	
AlCrFeCoNi	Selective electron beam melting (SEBM)	FCC + BCC	Phase evolution occurred during the preheating process	[134,135]
AlCrFeCoMnNi	LPBF	BCC (B2, A2)	B2 (Ni-Al rich) and A2 (Fe-Cr rich)	[136]
			Due to liquid-phase spinodal decomposition and cubic nature of the HEA	
Al_0.3_CoCrFeNi	LPBF	Supersaturated FCC phase	Fine columnar grains were present due to rapid solidification and anisotropic heat removal.	[139]
AlCoCrFeNiTi_0.5_	Laser-engineered net shaping (LENS)	2 BCC (B2, A2)	A fully equiaxed grain microstructure was exhibited rather than a columnar microstructure associated with alloys fabricated with AM.	[140]
AlCrCuFeNi	LPBF	2 BCC (B2, A2)	Unique columnar grains were present containing multiple ultrafine sub-grain structures.	[145]
AlCrFeNiV	LPBF	FCC	Rapid cooling rate and solidification resulted in the formation of sub-grains in every columnar grain and L1_2_ nano-phase.	[148]
AlCrFe_2_Ni_2_	LPBF	BCC	Columnar BCC of spinodal decomposed B2 and A2 structures was exhibited.	[149]
			Cracks were present at the intergranular site.	
FeCoCrNi	LPBF	FCC	After annealing at 1373 K, columnar grains and equiaxial grains were found to co-exist.	[150]
AlCoCrFeNi	Direct laser fabrication (DLF)	BCC (B2)	Intergranular needle-like and plate-like FCC phase precipitates and wall-shaped FCC phase precipitates were present along grain boundaries after aging at 800, 1000, and 1200 °C.	[151]
MoNbTaW	Direct energy deposition (DED)	BCC		[152]
Al_0.5_Cr_1.0_Mo_1.0_Nb_1.0_Ta_0.5_	SEBM	BCC	Two phases were present: TaMoNbCr and (TaMoNbCr)_Al_ solid solutions.	[153]
CoCrCuFeNiAl	LENS	BCC (B2, A2)	Dendritic grains were present.	[154,155]
			An ordered interface transition region was present between the two phases.	
AlCoCrFeNi_2.1_	LENS	Ordered FCC (L1_2_) + BCC	Co, Cr, and Fe stabilize L1_2_.	[156]
			L1_2_ and BCC are rich in nickel.	
Fe_38.5_Mn_20_Co_20_Cr_15_Si_5_Cu_1.5_	LPBF	FCC	Deformation-induced phase transformation of γ (FCC) to ε (HCP) occurred in the vicinity of microcracks.	[157]
CoCrFeNi	3D extrusion printing	FCC	There was complex structural evolution, from loosely packed oxide particles in the green body to fully-annealed, metallic CoCrFeNi.	[158]
AlCrFeMoV_x_ (x = 0 to 1)	LENS	BCC	The high solubility of V offers a broad range of solid solution strengthening of a compositionally complex but structurally simple BC matrix.	[158]
ZrTiVCrFeNi	LENS	C14 Laves phase (major) + α-Ti solid solution	The C14 Laves phase becomes stable on exposure to annealing and hydrogen influence.	[159]
6FeNiCoSiCrAlTi	Laser cladding	BCC	Equiaxed polygonal grains, discontinuous interdendritic segregation, and nano-precipitates are present.	[160]
MoFeCrTiW	Laser cladding	BCC	Cellular crystals are formed on which dispersion precipitates exist.	[161]
TiZrNbMoV	LENS	FCC (δTiH_x_-type) + BCC (NbH_∼__0.4_–type)	αZr-rich precipitates are present, in addition to the phases formed.	[162]
Al_0.5_FeCu_0.7_NiCoCr	Laser cladding	FCC + BCC + Al phases	A laser rapid cooling rate facilitates the formation of a simple structure and prohibits the formation of undesired intermetallic compounds.	[163]
TiZrNbHfTa	Laser metal deposition (LMD)	BCC	An equiaxed grain shape is present.	[164]
Al_0.5_CrMoNbTa_0.5_	Electron beam melting (EBM)	BCC	Intermetallic phases C14, C36, C15, and 6H are present.	[165]
Ni_6_Cr_4_WFe_9_Ti	LPBF	FCC	Tiny precipitates of an unknown phase are present.	[166]
FeCoCrNiC_0.05_	LPBF	FCC	Nano-scale Cr_23_C_6_-type carbides can precipitate under annealing conditions.	[167]

**Table 4:** Different strengthening mechanisms used to improve the mechanical properties of some HEAs processed by different fabrication methods.

HEA Composition	Observed Phase(s) through Different Processing Route(s)			Strengthening Mechanism in Respective Processing Route(s)			Effects on Mechanical Properties		
	Melting and Casting	MA + SPS	AM	Melting and Casting	MA + SPS	AM	Melting and Casting	MA + SPS	AM
CoCrFeNiMn	FCC [11,46]	FCC [192]	FCC + BCC [137,146,147]		Solid solution strengthening	Grain boundary strengthening		Compressive strength of 1987 MPa	Tensile strength of 601 MPa
								Hardness of 646 HV	
CoCrFeNiAl_0.3_	FCC [68,69]	FCC + BCC [112]	FCC [139]	Grain boundary strengthening	Solid solution strengthening	Dislocation hardening	UTS of 528 MPa	Compressive strength of 1907 MPa	YS of 730 MPa
							YTS of 275 MPa	Hardness of 625 HV	UTS of 896 MPa
CoCrFeNi		FCC + Cr_7_C_3_ [23]	FCC [150]		Grain boundary strengthening (470 HV), precipitation strengthening			Hardness of 580 HV	
AlCoCrCuFeNi	FCC + BCC [64]	FCC + BCC [189]	BCC [154]	Solid solution strengthening	Grain boundary strengthening, solid solution strengthening		Hardness of 515.5 HV (5.056 GPa)	Hardness of 8.13 GPa	
							Compressive strength of 1.82 GPa	Elastic modulus of 172 GPa	
TiZrNbMo_0.3_V_0.3_	BCC [193]		FCC + BCC [162]	Solid solution strengthening			Yield strength of 1312 MPa and 50% increase in plastic strain		
Ni_1.5_Co_1.5_CrFeTi_0.5_	FCC [48]	FCC [119]		Solid solution hardening	Grain boundary strengthening		YS of 896 MPa	Hardness of 442 HV0.3	
							Compressive strength of 1502 MPa	Tensile strength of 1384 MPa	
							Hardness of 515 HV	Elastic modulus of 216 GPa	
Al_0.7_FeCoCrNi_1.3_			FCC + BCC [172]			Precipitation strengthening by the B2 NiAl phase in an Fe-Cr-Ni matrix			A good compromise between hardness (280 HV) and strength
						Grain boundary precipitation of the Ni-Al-rich phase			
(FeCoNiCr)_94_Ti_2_Al_4_	FCC [168]			Precipitation hardening (327.7 MPa), dislocation hardening (274.5 MPa),			Accumulated yield strength of 645 MPa		
				grain boundary hardening (122.6 MPa)					
CuCr_2_Fe_2_NiMn	FCC [59]			Precipitation hardening of the *ρ* phase			Hardness of 450 HV		
FeCrNiCoMn	FCC [194]			Grain boundary strengthening			Increase in yield strength from 200 to 350 MPa		
Al_0.3_CrFe_1.5_MnNi_0.5_	FCC + BCC [56]			Precipitation hardening			Hardness of 800 HV		
Ni_2_CoCrFeNb_0.15_	FCC [170]			Precipitation strengthening (670 MPa), solid solution hardening (41.7 MPa)			Total yield strength of (954 MPa)		
							Ductility (27%)		
							Excellent yield strength–ductility combination		
Al_0.5_CrFeNiCo_0.3_C_0.2_		FCC + BCC [117]			Solid solution strengthening			Compressive strength of 2131 MPa	
								Hardness of 617 ± 25 HV	
CoCrFeNiMo_0.3_	FCC [195]			Precipitation hardening			Tensile strength of 1.2 GPa and good ductility of ∼19%		
FeCoCrNiMnTi_0.1_C_0.1_		FCC [196]			Grain boundary strengthening (61.3%),			Yield strength of 1652 MPa	
					precipitation strengthening (20.6%),			Hardness of 461 HV	
					dislocation strengthening (15.0%)				
Co_25_Ni_25_Fe_25_Al_7.5_Cu_17.5_		FCC [197]			Grain boundary strengthening,			Compressive yield strength of 1795 MPa	
					dislocation strengthening			Hardness of 454 HV	

## Data Availability

The data presented in this study are available on request from the corresponding author.
